# Early Adaptations to a Two-Week Uphill Run Sprint Interval Training and Cycle Sprint Interval Training

**DOI:** 10.3390/sports6030072

**Published:** 2018-07-27

**Authors:** Mykolas Kavaliauskas, John Jakeman, John Babraj

**Affiliations:** 1 School of Applied Sciences, Edinburgh Napier University, Edinburgh EH11 4BN, UK; 2 Department of Sport, Health Sciences and Social Work, Oxford Brookes University, Oxford OX3 0BP, UK; jjakeman@brookes.ac.uk; 3 Division of Sport and Exercise Sciences, Abertay University, Dundee DD1 1HG, UK; j.babraj@abertay.ac.uk

**Keywords:** high-intensity interval training, training adaptations, lactate, ventilator threshold

## Abstract

This study sought to compare early physiological and performance adaptations between a two-week cycle sprint interval training (SIT) and uphill run sprint training (UST) programs. Seventeen recreationally active adult males (age = 28 ± 5 years; body mass (BM) = 78 ± 9 kg) were assigned to either a control (*n* = 5), SIT (*n* = 6), or UST (*n* = 6) group. A discrete group of participants (*n* = 6, age = 33 ± 6 years, and body mass = 80 ± 9 kg) completed both training protocols to determine acute physiological responses. Intervention groups completed either a run or cycle peak oxygen uptake (VO_2_peak) test (intervention type dependent) prior to and following two weeks of training. Training comprised of three sessions per week of 4 × 30-s “all-out” sprints with a four-minute active recovery between bouts on a cycle ergometer against 7.5% of body mass in the SIT group and on a 10% slope in the UST group. The VO_2_peak values remained unchanged in both training groups, but time-to-exhaustion (TTE) was significantly increased only in the UST group (pre—495 ± 40 s, post—551 ± 15 s; *p* = 0.014) and not in the SIT group (pre—613 ± 130 s, post—634 ± 118 s, *p* = 0.07). Ventilatory threshold (VT) was significantly increased in both training groups (SIT group: pre—1.94 ± 0.45 L·min^−1^, post—2.23 ± 0.42 L·min^−1^; *p* < 0.005, UST group: pre—2.04 ± 0.40 L·min^−1^, post—2.33 ± 0.34 L·min^−1^, *p* < 0.005). These results indicate that UST may be an effective alternative to SIT in healthy individuals.

## 1. Introduction

Cycle sprint interval training (SIT) consisting of repeated brief “all-out” cycle sprints interspersed with recovery periods offers a time-efficient alternative to traditional endurance training [1]. A commonly studied SIT protocol involves 30-s Wingate tests against 7.5% of body mass repeated four to six times separated by 4 min of recovery [2]. For example, six sessions of SIT performed over two weeks have been shown to improve skeletal muscle oxidative metabolism and cycling time to exhaustion in recreationally active individuals [3]. Seven weeks of progressive SIT in healthy men significantly increased glycolytic and oxidative muscle enzyme activity, maximum short-term power output, and maximal oxygen uptake (VO_2_max) [4]. Similarly, aerobic and anaerobic adaptations as demonstrated by improvements in a 5-km cycling time trial, VO_2_max, peak, and average power output have been found after two weeks of SIT in healthy, young adults [5].

Although SIT offers a low-volume training paradigm with significant health and performance benefits, previous studies mainly used specialized cycle ergometers to control the intensity of the exercise [3,4,5,6]. While cycle ergometers are accurate, they are not always ecologically valid and may be relatively costly to acquire. The uphill sprint training (UST), which is also called running SIT, may offer a viable option in the training prescription “menu” to elicit training adaptations in a short time frame without needing access to any specialized equipment. However, it may not always be possible to complete the UST outdoor where weather and/or a suitable incline cannot be controlled. Since there are both advantages and disadvantages to these training approaches, it would be of use to understand to what extent these approaches can be used interchangeably to allow practitioners the scope to select the most appropriate training approach for their need.

Previous research has demonstrated that UST is an effective training modality in a range of exercise programs and athletic activities. For example, eight weeks of UST has been shown to increase VO_2_max and insulin sensitivity and reduce plasma low density lipoprotein-cholesterol in healthy young participants [7]. Similarly, a more recent study by Willoughby et al. [8] found that four weeks of UST improves cardiorespiratory and anaerobic fitness in young and middle-aged adults. In addition, the efficacy of UST has been demonstrated in athletic populations including the semi-professional male soccer players [9], semi-professional female field hockey players [10], and well-trained distance runners [11]. While both sprinting protocols appear to lead to similar improvements in cardiorespiratory fitness in non-athletic populations (cycling—6.2–7.8% [12,13], running—3.9–11.5% [9,10], no studies have directly measured early physiological responses between SIT and UST in healthy, recreationally-trained male adults.

Therefore, the primary aim of this study was to compare early physiological and performance adaptations, which is represented by peak oxygen uptake (VO_2_peak), time-to-exhaustion (TTE), and the ventilatory threshold (VT) following six sessions of SIT and UST performed over two weeks. The secondary aim was to determine acute physiological responses following both protocols to help understand mechanisms underpinning the training adaptations. We hypothesized that six sessions of UST would lead to similar early physiological adaptations compared to SIT.

## 2. Materials and Methods

### 2.1. Participants

Seventeen healthy, recreationally active men (minimum 3 sessions per week of 45 min with moderate intensity exercise) participated in the training study. Participants were randomly allocated to a control group (CG), sprint interval training (SIT) group, or an uphill sprint training (UST) group. A discrete group (DG) of 6 participants completed both training protocols to determine acute physiological responses. The characteristics of the participants are presented in Table 1.

All groups were asked to continue with their regular daily activities and training programs throughout the study period. Participants were also asked to refrain from any vigorous exercise 24 h before each test. The participants were informed of the experimental protocol both verbally and in writing before giving informed consent. The study protocol was approved by the Abertay University Ethics Committee and conducted in accordance with the Declaration of Helsinki.

### 2.2. Procedures

#### 2.2.1. Baseline Testing

After reporting to the Human Performance Laboratory, the UST group completed only the run VO_2_peak test and the SIT group completed only the cycle VO_2_peak test. The control group completed both run and cycle VO_2_peak tests in a randomized fashion separated by a minimum of 48 h.

#### 2.2.2. Run VO_2_Peak 

Participants performed an incremental treadmill test to volitional exhaustion on a motorized treadmill (H/P/Cosmos Mercury, Nussdorf-Traunstein, Germany) to determine VO_2_peak via breath by breath analysis (Metalyzer^®^3B gas analyzer, Cortex, Leipzig, Germany), which was described by Harling et al. [14]. In addition, time-to-exhaustion (TTE) was recorded using a Quantum 5500 stop clock (EA Combs Ltd., London, UK). Participants performed a standardized warm-up on a treadmill for 5 min at 7.5 km·h^−1^. The incremental test then began at 10 km·h^−1^ with the speed increased by 1 km·h^−1^ every minute until volitional exhaustion. At the end of the test, participants walked on the treadmill for 5 min at 5 km·h^−1^ at a 0% inclination. The VO_2_peak calculated as the highest oxygen consumed over a 30-s period and ventilatory threshold was calculated using the V-slope method [15]. 

#### 2.2.3. Cycle VO_2_Peak

Participants performed an incremental cycling test to volitional exhaustion to determine the VO_2_peak using breath by breath analysis (Metalyzer^®^3B gas analyzer, Cortex, Leipzig, Germany). The test was designed to produce a similar time to exhaustion as the run VO_2_peak test described above. The TTE was recorded using a Quantum 5500 stop clock (EA Combs Ltd., London, UK). The participants performed a 5 min warm up cycling at 60 W (Monark 894E Peak bike, Monark Exercise AB, Vansbro, Sweden). The test then began with the participant cycling at 60 W for 1 min and the intensity increased by 25 W every minute until volitional exhaustion or the participant could not maintain a cadence of 60 r·min^−1^. During the test, participants could pedal faster than 60 r·min^−1^. At the end of the test, participants cycled for 5 min at 30 W. The VO_2_peak calculated as the highest oxygen consumed over a 30-s period and the ventilatory threshold was calculated using the V-slope method [15]. Both VO_2_peak tests were repeated after two weeks for the control group and three days after the completion of training for the intervention groups. All tests were performed within 2 h of the same time of the day.

#### 2.2.4. Sprint Interval Training Protocol

The SIT protocol was similar to the protocol used previously [16]. Six sprint interval sessions were spread over 14 days with a minimum of 24 h of rest between sessions. Each training session consisted of 4 × 30-s “all-out” cycling efforts against 7.5% of body mass with 4 min of active recovery between sprints (1:8 work-to-rest ratio). Resistance was automatically applied to the cycle ergometer (Monark 894E Peak bike, Monark Exercise AB, Sweden) once the participant was cycling at 110 r·min^−1^, which initiated the start of the 30-s cycle sprint. During recovery, participants remained on the bike and cycled at a low cadence (<50 r·min^−1^) without resistance. Peak and average power output was automatically calculated for each sprint in the six training sessions using the Monark Anaerobic Test Software version 2.24.2 (Monark Exercise AB, Vansbro, Sweden).

#### 2.2.5. Uphill Sprint Training Protocol

The UST protocol consisted of six uphill sprint sessions spread over 14 days with a minimum of 24 h of rest between sessions. Similar to previous studies [11,17], each training session consisted of 4 × 30-s “all-out” uphill sprint efforts on a 10% slope. During a 4-minute recovery, subjects walked back down the hill to the starting position. Average power output during the uphill sprint was calculated using the following equations as described by di Prampero [18].

Work = Potential Energy = m × g × d × sinθ where m is the participants mass in kg, g is the force of gravity, d is the distance covered in 30 s, and θ is the angle of the hill.

Power = W/t where W is the work done and t is the time duration of the sprint.

#### 2.2.6. Acute Responses to both Training Protocols

Six participants from the discrete group performed 2 × 30-s “all-out” efforts using both sprint interval and uphill sprint training protocols in a randomized order on different days separated by at least 24 h. Heart rate (Polar Electro, Kempele, Finland), VO_2_, and VCO_2_ (MetaMax^®^3B gas analyser, Cortex, Leipzig, Germany) were recorded continuously throughout the sprint and each 4 min recovery period averaged over 5 s. 

#### 2.2.7. Lactate Measurement

Fingertip blood samples were taken immediately upon completion of each sprint and compared to a sample taken prior to the training session to analyze blood lactate concentration. The skin was punctured using an Accu-check single use lancet (Roche Diagnostics, Burgess Hill, UK) and pressure applied to the finger to draw the capillary blood. The initial drop was discarded and the second drop was taken for lactate analysis using the Lactate Pro blood lactate meter (Arkray Inc., Kyoto, Japan). A cotton pad was placed on the incision and pressure applied until bleeding had stopped.

### 2.3. Statistical Analysis

Data are expressed as a mean ± standard deviation. Area under the curve for heart rate (HR), VO_2_, and VCO_2_ was calculated using the standard trapezoid rule [19]. The Shapiro-Wilk test was used to determine whether data were normally distributed and a paired sample *t*-test was used to compare the acute and training effect within a group. An unpaired *t*-test was used to compare between groups [7]. The null hypothesis was rejected at the 5% level (*p* < 0.05). Effect size between the groups was calculated using the method of Morris and DeShon for repeated measure design to allow for the correction for different sample sizes and pre-test values [20]. The effect size for the acute response was calculated as Cohen’s *d*, which allows for measuring the difference between the groups in terms of their common standard deviation. For both, the effect size was defined as follows: *d* < 0.2 trivial effect, 0.2–0.5 small effect, 0.6–1.1 moderate effect, and 1.2–1.9 as a large effect [21].

## 3. Results

### 3.1. Training Results

#### 3.1.1. VO_2_Peak 

At baseline, the VO_2_peak was similar between training groups (SIT: 49 ± 7 mL·kg^−1^·min^−1^, UST: 48 ± 4 mL·kg^−1^·min^−1^, *p* > 0.05) and did not significantly change in both groups called SIT (pre: 49 ± 7 mL·kg^−1^·min^−1^, post: 49 ± 7 mL·kg^−1^·min^−1^, *p* > 0.05) and UST (pre: 48 ± 4 mL·kg^−1^·min^−1^, post: 50 ± 6 mL·kg^−1^·min^−1^, *p* > 0.05) after two weeks of training. However, there was a small effect size between groups with a greater change in UST (*d* = 0.34).

#### 3.1.2. Time-to-Exhaustion

**Figure 1:**
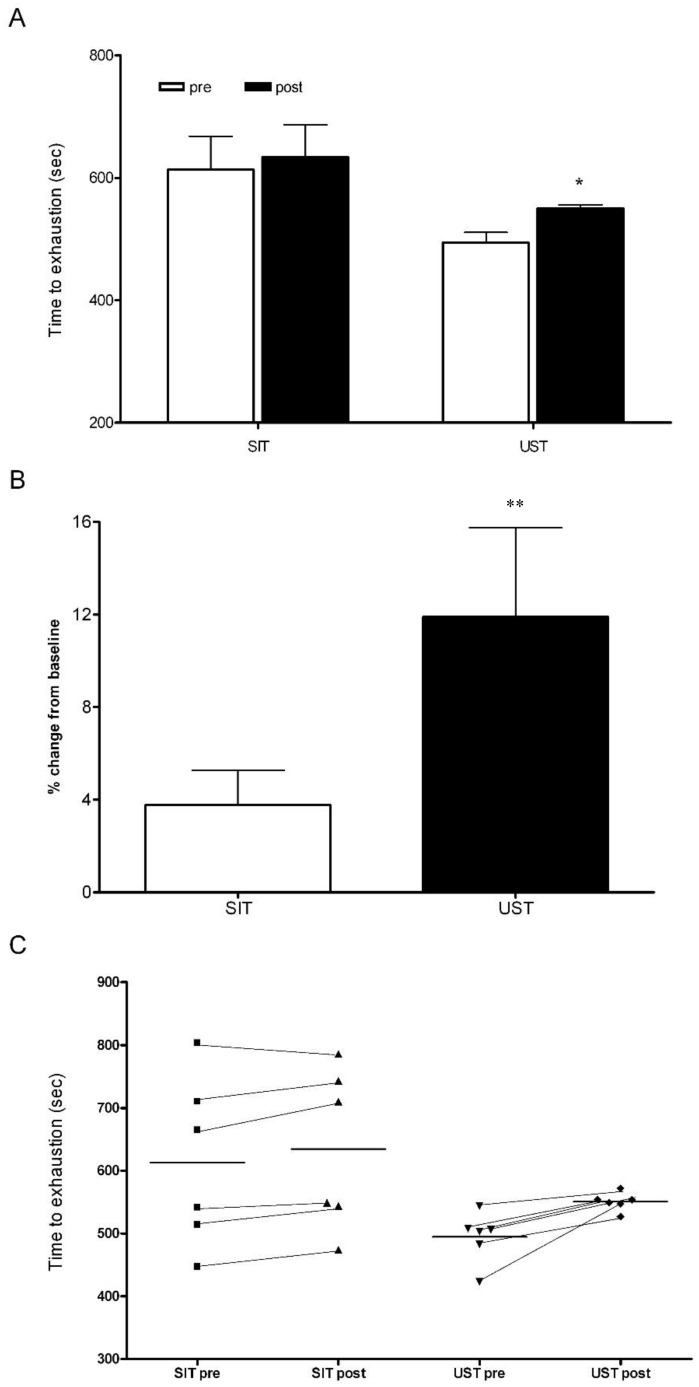
Absolute percentage and individual changes in time-to-exhaustion in SIT and UST groups. (**A**) Absolute changes pre-SIT and post-SIT and UST, * *p* < 0.05 pre-compared to post; (**B**) Percentage change from the baseline in SIT and UST groups, ** *p* < 0.05 SIT compared to UST; (**C**) Individual changes in time-to-exhaustion pre-SIT and post-SIT and UST.

There was no significant difference in the TTE for the cycling and running protocols in the control group (running TTE: 426 ± 71 s, cycling TTE: 515 ± 102 s, *p* > 0.05). There were also no significant changes in the TTE during the cycling and running protocols in the control group after two weeks (running TTE: 426 ± 71 s vs. 441 ± 94 s, *p* > 0.05, cycling TTE: 515 ± 102 s vs. 537 ± 101 s, *p* > 0.05). At baseline, TTE was similar between training groups (SIT: 613 ± 135 s, UST: 495 ± 40 s, *p* > 0.05, Figure 1). Following 2 weeks of training, the TTE had increased by ~3% in the SIT group and ~11% in the UST group (SIT: 613 ± 135 s vs. 634 ± 118 s, *p* = 0.07, UST: 495 ± 40 s vs. 551 ± 15 s, *p* = 0.014, Figure 1). The magnitude of the change in TTE was significantly different between the training groups (SIT: 3 ± 5%, UST: 11 ± 9%, *p* = 0.04). There was a small effect size between training groups with a greater change in UST (*d* = 0.34) and a large effect size between the control group and the UST group (*d* = 0.71).

#### 3.1.3. Ventilatory Threshold

**Figure 2:**
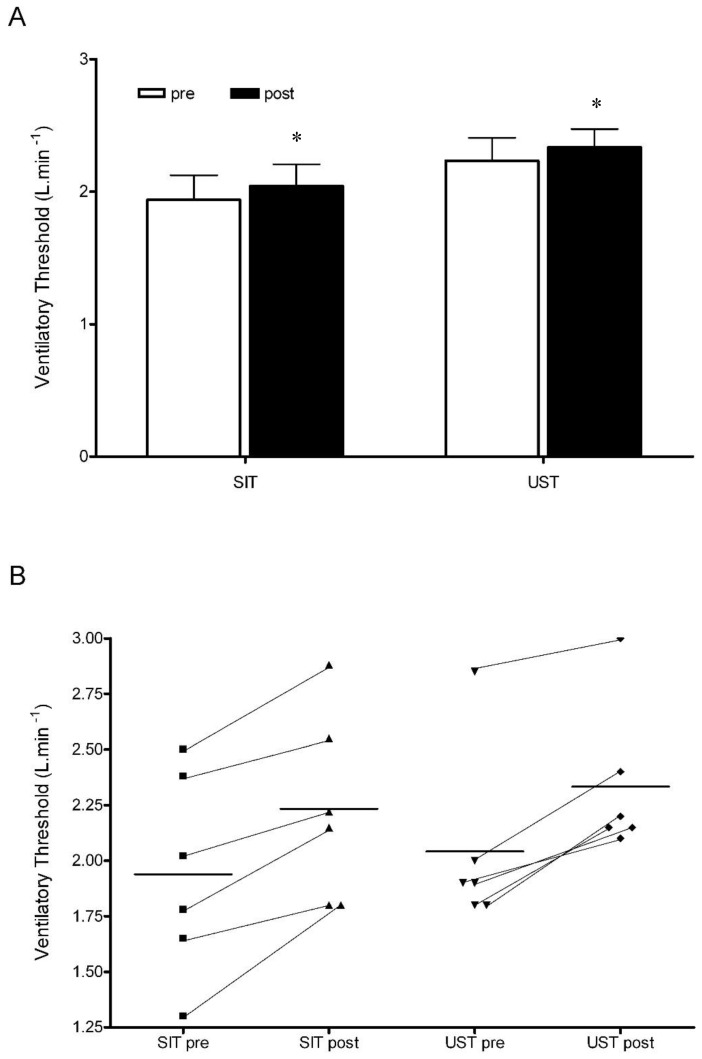
Absolute and individual changes in the ventilatory threshold in SIT and UST groups, (**A**) Ventilatory threshold pre-SIT and post-SIT and UST, * *p* < 0.05 pre compared to post; (**B**) Individual changes in ventilatory threshold pre-SIT and post-SIT and UST.

At baseline, there was no significant difference in the VT for either of the training group (SIT: 1.94 ± 0.45 L·min^−1^, UST: 2.04 ± 0.40 L·min^−1^, *p* > 0.05, Figure 2). In both training groups, the VT was significantly increased after two weeks of training (SIT: pre—1.94 ± 0.45 L·min^−1^, post—2.23 ± 0.42 L·min^−1^, *p* < 0.005; UST: pre—2.04 ± 0.40 L·min^−1^, post—2.33 ± 0.34 L·min^−1^, *p* < 0.005; Figure 2). There was no significant difference in the magnitude of change between groups (SIT: 16 ± 11%, UST: 15 ± 6%; *p* > 0.05).

#### 3.1.4. Average Power

In both groups, the average power produced was similar across all sessions (Table 2). The power drop between sprint 1 and 4 was significantly altered after UST but not SIT (UST session 1: 26 ± 4%, session 6: 14 ± 4%, *p* = 0.001, SIT session 1: 23 ± 11%, session 6: 17 ± 5%, *p* = 0.18). There was a large effect size for the power drop between the two groups with a greater improvement in UST (*d* = 0.70).

### 3.2. Acute Responses of Training

#### 3.2.1. Blood Lactate

**Figure 3:**
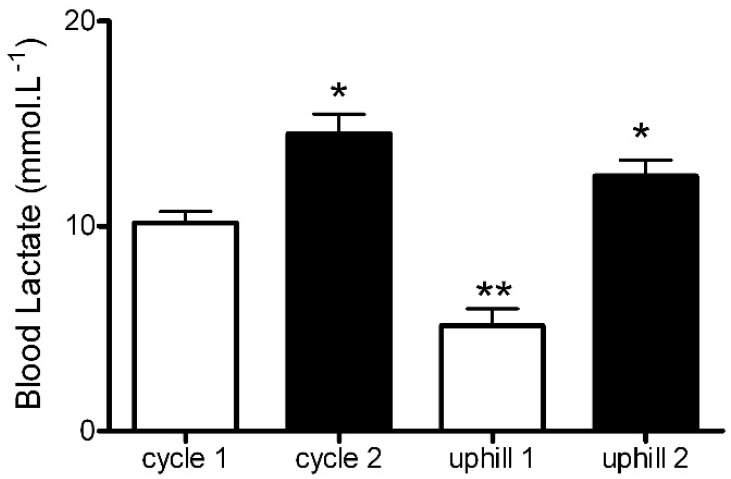
Blood lactate concentration following uphill run sprints and cycle sprints. * *p* < 0.01 sprint 1 compared to sprint 2. ** *p* < 0.001 cycle sprint 1 compared to uphill sprint 1.

Blood lactate was similar between groups at the baseline (SIT: 1.9 ± 0.4 mmol·L^−1^, UST: 1.9 ± 0.2 mmol·L^−1^) and significantly higher after each sprint when compared to the baseline (SIT sprint 1: 10.2 ± 1.2 mmol·L^−1^, sprint 2: 14.1 ± 1.7 mmol·L^−1^; *p* < 0.01, UST sprint 1: 5.1 ± 2.4 mmol·L^−1^, sprint 2: 12.5 ± 2.2 mmol·L^−1^, *p* < 0.001, Figure 3). The rise in blood lactate was significantly greater after the first SIT sprint when compared to the first UST sprint (SIT sprint 1: 10.2 ± 1.2 mmol·L^−1^ vs. UST sprint 1: 5.1 ± 2.4 mmol·L^−1^, *p* < 0.001, *d* = 2.70, Figure 3). However, this difference was not significant following sprint 2, but there was still a large effect between the groups (SIT sprint 2: 14.1 ± 1.7 mmol·L^−1^ vs. UST sprint 2: 12.5 ± 2.2 mmol·L^−1^, *p* > 0.05, *d* = 0.81, Figure 3).

#### 3.2.2. Heart Rate, VO_2_, and VCO_2_

**Figure 4:**
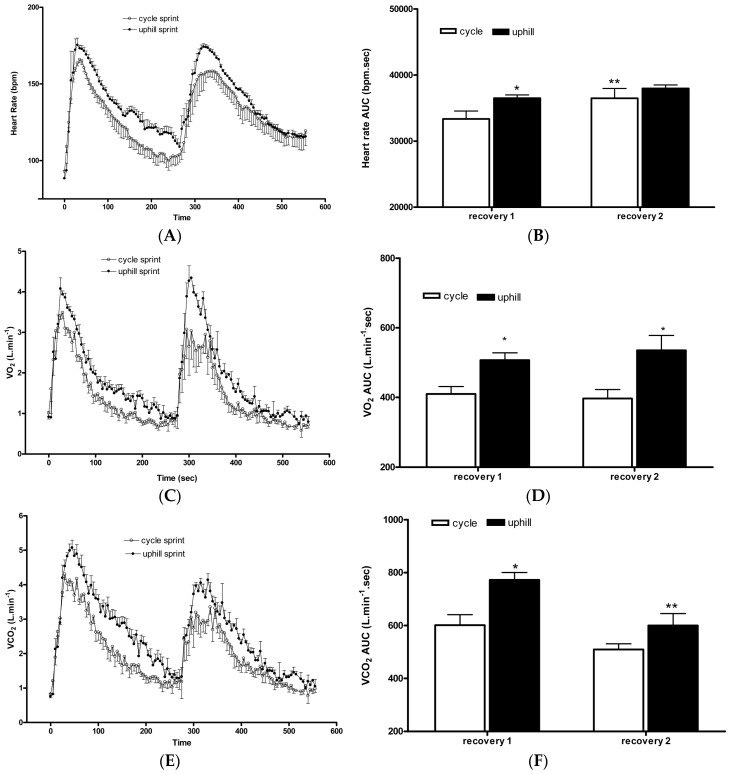
Changes in heart rate, VO_2_, and VCO_2_ during sprints and recovery. (**A**) Heart rate response; (**B**) Heart rate area under the curve, * *p* < 0.05 cycle sprint 1 compared to uphill sprint 1, ** *p* < 0.05 sprint 1 compared to sprint 2; (**C**) VO_2_ response. (**D**) VO_2_ area under the curve, * *p* < 0.05 sprint 1 compared to sprint 2; (**E**) VCO_2_ response; F: VCO_2_ area under the curve, * *p* < 0.001 cycle sprint 1 compared to uphill sprint 1, ** *p* < 0.001 sprint 1 compared to sprint 2.

Heart rate increased during both SIT and UST protocols and remained elevated above resting during recovery (Figure 4A). There was no difference in the heart rate area under the curve (AUC) during both SIT and UST. However, sprint 1 in the UST group had a significantly greater AUC during recovery compared to sprint 1 in the SIT group (UST sprint 1: 36,510 ± 1119 beats vs. SIT sprint 1: 33,373 ± 2899 beats, *p* < 0.05, *d* = 1.43, Figure 4B). Heart rate AUC was significantly greater following sprint 2 compared to sprint 1 in the SIT group but not following sprint 2 in the UST group with a moderate effect between groups (SIT sprint 1: 33,373 ± 2899 beats vs. sprint 2: 36,496 ± 2954 beats; *p* < 0.05, UST sprint 1: 36,510 ± 1119 beats vs. sprint 2: 37,976 ± 1064 beats, *p* > 0.05, *d* = 0.67, Figure 4B).

VO_2_ increased during both SIT and UST protocols and remained elevated above a resting heart rate during recovery (Figure 4C). There was no difference in VO_2_ AUC during SIT and UST. However, the UST group had a greater AUC during the recovery of sprint 1 (*d* = 1.96) and 2 (*d* = 1.94) compared to sprint 1 and 2 of the SIT group (SIT sprint 1: 409 ± 53 mL·kg^−1^ vs. UST sprint 1: 507 ± 47 mL·kg^−1^, *p* < 0.05, SIT sprint 2: 397 ± 52 mL·kg^−1^ vs. UST sprint 2: 535 ± 86 mL·kg^−1^, *p* < 0.001, Figure 4D). VO_2_ AUC was not different between sprint 2 and sprint 1 in the SIT group or sprint 2 and sprint 1 in the UST group (*p* > 0.05, Figure 4D).

VCO_2_ increased during both SIT and UST protocols and remained elevated above resting during recovery (Figure 4E). There was no difference in VCO_2_ AUC during SIT and UST. However, the UST group had a greater AUC during the recovery of sprint 1 compared to sprint 1 of the SIT group (SIT sprint 1: 601 ± 97 mL·kg^−1^ vs. UST sprint 1: 772 ± 64 mL·kg^−1^, *p* < 0.001, *d* = 2.08, Figure 4F). VCO_2_ AUC was significantly greater following sprint 1 when compared to sprint 2 only in the UST group but not in the SIT group with a large effect between groups (SIT sprint 1: 601 ± 97 mL·kg^−1^ vs. sprint 2: 509 ± 43 mL·kg^−1^, *p* > 0.05, UST sprint 1: 772 ± 64 mL·kg^−1^ vs. sprint 2: 600 ± 90 mL·kg^−1^, *p* < 0.001, *d* = 1.29, Figure 4F).

## 4. Discussion

While SIT has been shown to be an effective training modality for performance and health benefits in tightly controlled laboratory-based studies, it is not necessarily user-friendly. In the present study, we demonstrate the effectiveness of UST on a 10% incline to induce aerobic adaptations, which is represented by peak oxygen uptake (VO_2_peak), time-to-exhaustion (TTE), and the ventilatory threshold (VT) that are similar in magnitude to those seen with SIT. In addition, different acute physiological responses to both training modalities are presented.

### 4.1. Training Adaptations

#### 4.1.1. VO_2_Peak

There were no improvements in VO_2_peak over two weeks with either training protocol. This is similar to previous studies that found no change in VO_2_peak following six SIT sessions performed over two weeks in eight recreationally active participants [3,22]. Conversely, others have shown a mean improvement between 6.3% to 9.3% in VO_2_peak following only two weeks of SIT in young, active adults [5,23]. Two recent meta-analyses further supported the effectiveness of a ‘traditional’ SIT protocol on VO_2_max improvement by demonstrating a likely moderate to large effect (6.2% to 7.8%) [12,13]. The differences in findings between studies can be attributed to a number of training parameters with the modifying effects on the magnitude of VO_2_max. These include the maximum number of sprint repetitions in a training session, sprint duration, number of training sessions, work-to-rest ratios, baseline VO_2_max, and training duration [12,13].

Contrasting effects on VO_2_max have also been reported following running SIT protocols. For example, Sandvei et al. [7] demonstrated a 5.3% improvement in VO_2_max in healthy, young participants following an eight-week 30 s progressive uphill (inclination 5% to 8%) sprinting protocol with a 3 min rest between each sprint. Additionally, MacPherson et al. [24] showed that running SIT, which consisted of four to six bouts of “all-out” 30-s sprints with 4 min of recovery performed three times per week for six weeks, increased VO_2_max by 11.5% in healthy, recreationally active participants. In contrast, Ferley et al. [11] found no improvements in VO_2_max after six weeks of UST in already well-trained participants (VO_2_max—63.3 ± 8.0 mL·kg^−1^·min^−1^). 

No changes in VO_2_peak in the current study suggests that a minimum cumulative training volume required for cardiorespiratory fitness improvement has not been reached in either training group. Therefore, more studies are required to assess the effects of various training parameters and their interaction on the magnitude and time course of training-induced physiological adaptations following SIT and UST. 

#### 4.1.2. TTE

Six sessions of UST performed over two weeks resulted in an 11% improvement in TTE compared to a 3% increase in the SIT group despite no changes in the VO_2_peak in both groups (Figure 1). The magnitude of the change in TTE was significantly larger in the UST group than in the SIT group (Figure 1). 

Similar to the findings of the current study, Ferley et al. [11] also reported no changes in the VO_2_max but did report a significant improvement of 31.7% during a functional TTE running test at the speed associated with VO_2_max in response to a six-week UST in well-trained runners. A significant improvement in TTE following UST can be attributed to significantly greater aerobic metabolic demands as demonstrated by a higher heart rate (Figure 4B), VO_2_ (Figure 4D), and VCO_2_ (Figure 4F) values during the recovery from the sprint when compared to the SIT. It is important to mention that the differences in the TTE results between the two training groups may also be due to a relatively large variability in the SIT group, which is demonstrated by a high SD (Figure 1). Future studies should assess the effects of sprint training on TTE using a different testing protocol. For example, Burgomaster et al. [3] have reported a two-fold improvement in TTE at ~80% VO_2_max following six sessions of SIT when using a continuous cycle protocol. Therefore, a continuous TTE cycle protocol at a fixed percentage of VO_2_max may be a more sensitive measure for detecting improvements in fatigability than in incremental protocols. Alternatively, short time-trials (TT) have been shown to have a higher degree of ecological validity and a lower coefficient of variation (CV) scores for performance compared to TTE [25].

Nevertheless, relatively high levels of VO_2_, heart rate, and ventilation, averaging above 80% of estimated maximal values, have been previously reported during and immediately after repeated SIT bouts in young, recreationally active, healthy adults [26]. This shows an increasing reliance on aerobic metabolism with each subsequent bout. The primary mechanism of adaptation to SIT involves enhancement of the supply and utilization of aerobic energy production [27]. Our results show that, compared to SIT, UST elicits even greater relative aerobic metabolic and cardiovascular responses, which subsequently leads to peripheral changes that may have an effect on muscle fatigability. As demonstrated in Table 2, average power production across all four sprints was significantly altered in the UST but not in the SIT group. The period after training both groups showed a different average power output profile between sprint 1–4. Specifically, except in session 1, the UST group demonstrated a lower absolute power drop-off between sprint 1–4, which occurred largely due to an improvement in power production in sprint 4 and little changes in sprint 1. Yet, the SIT group improved the average power production in both sprint 1 and 4, but the absolute drop-off still remained higher than in the UST group (Table 2). 

#### 4.1.3. VT

Our results demonstrate that the ventilatory threshold was significantly improved following two weeks of SIT and UST (Figure 2). The values for VT are similar to those reported previously for moderately active individuals [15]. VT has been shown to relate to lactate accumulation [15]. Following six sessions of the progressive 30-s “all-out” SIT programmer, it has been shown that skeletal muscle lactate accumulation is reduced during a two stage submaximal cycle test [3] and during a 30-s maximal sprint [28]. The decrease in lactate accumulation in skeletal muscle could be due to a decreased rate of glycogenolysis after SIT [3] or due to an increased activity of pyruvate dehydrogenase (PDH), which allows for an increased use of pyruvate in oxidative metabolism [29]. Furthermore, there is an increase in skeletal muscle MCT1 and MCT4 content after one and six weeks of 30-s sprint SIT [16], which may be linked with an increased skeletal muscle lactate uptake [30]. 

From a practical point of view, VT provides a better aerobic fitness index for sustainable submaximal work and competitive endurance performance than the VO_2_peak [15]. Therefore, depending on their personal preference, practitioners and athletes can use either of these training modalities to improve VT and sporting performance.

### 4.2. Acute Responses

In the current study, the cardiovascular demand of UST and SIT was the same during the 30-s sprints (Figure 4A), which was supported by the similar average power production in each training session (Table 2). However, during the recovery phase after the first sprint, the cardiovascular demand was greater following the UST when compared to the SIT (Figure 4B). Following longer duration maximal and submaximal running and cycling heart rate has been shown to be greater for running exercise [31]. It was demonstrated that there is a lower venous return following cycling when compared to running, which results in a lower cardiac output and stroke volume [32]. Moreover, the ‘muscle pump’ efficiency is greater in running compared to cycling due to the erect position during running and the type of contraction performed [32]. A greater cardiovascular demand during the recovery phase in the UST group can be linked to a larger improvement in the TTE when compared to the SIT group (Figure 1).

Blood lactate concentration represents a balance between lactate production and lactate use. It has been proposed that lactate can be shuttled around the body from the site of production to other tissues or non-exercising skeletal muscle [33]. We observed that blood lactate accumulation following the first uphill sprint was significantly lower than accumulations following the first SIT bout (Figure 3). Limb blood flow has been shown to be greater after running than cycling [32] and, as such, lactate produced during the UST may have been more effectively shuttled to other tissues and non-exercised skeletal muscle than during the SIT. Additionally, a significantly greater oxygen demand (Figure 4D) and lower blood lactate concentration (Figure 3) following the first uphill sprint suggests that there may be a greater aerobic contribution during uphill sprinting when compared to SIT. This may have important practical considerations when designing training programs with the primary goal of enhancing aerobic adaptations.

## 5. Study Limitations

One of the limitations of the current study is a small sample size. Second, participants’ training background was not controlled, but the randomization process should limit the possibility of it having an effect on the current findings. In addition, training protocols were only matched for the total duration of work (2 min), recovery (16 min), and the work-to-rest ratio (1:8), but not for the total work. However, average power outputs presented in Table 2 suggest that the external training load was similar in both training groups. Future studies should evaluate the magnitude of physiological adaptations when the total work is constant between the two different training groups. Therefore, there is a need for larger and longer studies to compare adaptations in response to the UST and SIT protocols. 

## 6. Conclusions

In conclusion, we demonstrate that UST on a 10% incline results in similar training adaptations compared to an SIT protocol. From a practical point of view, this offers a free ecologically valid training modality to the ‘traditional’ laboratory-based SIT method. The mechanisms underpinning the training adaptations for this type of exercise still need to be elucidated. However, improvements in lactate metabolism are similar between both training regimens. The metabolic demands of a single training session are greater following the UST protocol with greater VO_2_, VCO_2_, and heart rate during the recovery from the sprint. However, these higher metabolic demands post-exercise were not related to increased blood lactate accumulation following uphill sprinting.

## Figures and Tables

**Table 1:** Characteristics of all participants (mean ± standard deviation).

Characteristic	CG (*n* = 5)	SIT (*n* = 6)	UST (*n* = 6)	DG (*n* = 6)
Age (years)	27 ± 4	32 ± 7	25 ± 5	33 ± 6
Body Mass (kg)	77 ± 9	74 ± 8	84 ± 9	80 ± 9
BMI (kg·m^−2^)	25 ± 4	23 ± 2	26 ± 3	25 ± 3

**Table 2:** Average power (W·kg^−1^) production and percentage drop-off in power between sprint 1 and 4 in all training sessions in both training groups.

Training	Sprint 1 Mean Power (W·kg^−1^)	Sprint 2 Mean Power (W·kg^−1^)	Sprint 3 Mean Power (W·kg^−1^)	Sprint 4 Mean Power (W·kg^−1^)	% Drop-Off between Sprint 1–4
SIT					
Session 1	7.7 ± 0.8	7.2 ± 0.5	6.5 ± 0.5	5.9 ± 0.8	23
Session 2	8.0 ± 0.7	7.4 ± 0.7	6.4 ± 0.8	6.3 ± 0.7	21
Session 3	8.0 ± 1.0	7.3 ± 0.6	6.6 ± 0.5	6.2 ± 0.7	23
Session 4	7.9 ± 0.9	7.3 ± 0.6	6.8 ± 0.8	6.5 ± 0.7	18
Session 5	7.9 ± 0.9	7.5 ± 0.5	6.8 ± 0.8	6.5 ± 0.6	18
Session 6	8.1 ± 0.9	7.6 ± 0.7	6.9 ± 0.6	6.7 ± 0.5	17
UST					
Session 1	7.4 ± 0.9	6.5 ± 0.9	5.6 ± 0.9	5.5 ± 0.6	26
Session 2	7.3 ± 1.0	6.6 ± 0.9	6.0 ± 1.0	6.0 ± 0.9	18
Session 3	7.0 ± 0.9	6.6 ± 1.0	6.1 ± 1.0	6.1 ± 0.8	13
Session 4	7.1 ± 1.0	6.5 ± 0.9	6.0 ± 0.8	5.8 ± 0.8	18
Session 5	7.1 ± 0.8	6.6 ± 0.9	6.2 ± 1.0	6.1 ± 0.8	14
Session 6	7.1 ± 0.8	6.5 ± 0.9	6.1 ± 1.0	6.1 ± 0.8	14
